# Reducing the Health Burden of HPV Infection Through Vaccination

**DOI:** 10.1155/IDOG/2006/83084

**Published:** 2006

**Authors:** David Soper

**Affiliations:** Department of Obstetrics and Gynecology, Medical University of South Carolina, Charleston, SC 29425, USA

## Abstract

Human papillomavirus (HPV), a sexually transmitted infection and the etiologic cause of genital warts and cervical cancer, is highly prevalent in sexually active men and women. Although cervical screening procedures have significantly reduced the disease burden associated with HPV infection, they are expensive and abnormal results cause significant emotional distress. Therefore, prevention may be an effective strategy for reducing the economic, psychosocial, and disease burden of HPV infection. Multivalent vaccines are now in clinical development. A bivalent vaccine that protects against HPV 16 and 18, and a quadrivalent vaccine which protects against HPV types 6, 11, 16, and 18, have been shown to significantly reduce the occurrence of incident and persistent HPV infections in phase 2 clinical trials; phase 3 trials are currently underway. HPV vaccines will be most effective when administered prior to initiation of sexual activity, and vaccination campaigns should aggressively target preadolescent and adolescent populations.

## SIGNIFICANCE OF HPV INFECTION

**Figure 1:**
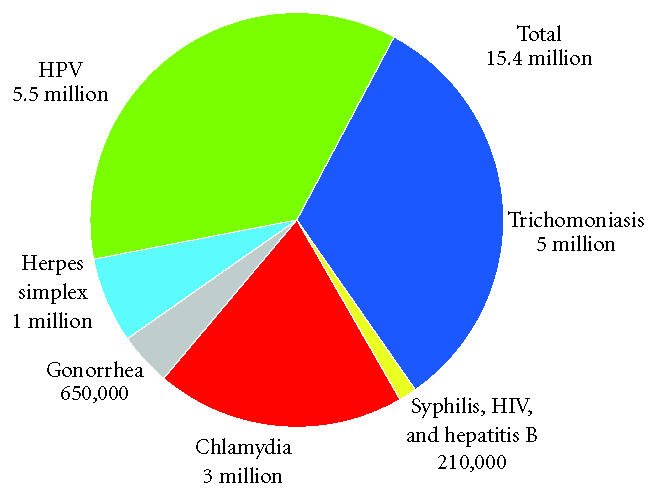
Estimated incidence of sexually transmitted infections in the United States [1].

Human papillomavirus (HPV) is the most common newly acquired sexually transmitted infection (STI) in the United States, with an estimated 20 million people infected [1]. Furthermore, incidence of HPV infection has increased during the past two decades, with approximately 6.2 million newly diagnosed cases annually [1, 2] (Figure 1). HPV infection has a very high prevalence rate in adolescent girls and young women. One study showed that 36% of women 25 years of age or younger are HPV-positive compared with less than 3% of women 45 years of age and older [3]. HPV is the etiologic agent of several genital epithelial lesions including genital warts (condylomata acuminata), cervical intraepithelial neoplasia (CIN), and cervical cancer. Consequently, HPV is a major public health burden.

More than 100 different types of HPV have been identified [4, 5] (Table 1). Low-risk types, HPV 6 and HPV 11, are the most common types implicated in causing genital warts [6]. Furthermore, due to their ability to cause low-grade cervical lesions, infection with low-risk HPV types is often associated with abnormal Papanicolaou (Pap) test results [7]. Although genital warts are medically benign, and low-grade CIN can spontaneously regress, diagnosis of genital warts or an abnormal Pap smear result can cause emotional distress and impose an economic burden [8].

In contrast to infection with HPV types 6 and 11, infection with high-risk HPV types 16 and 18 can lead to anogenital cancers. HPV types 16 and 18 cause 70% of all cases of cervical cancer, with half of all cervical cancers caused by type 16 alone [4]. Persistent infection with high-risk HPV types is implicated in 99.7% of cervical cancers [9]. Preventing infection with the most common low-risk and high-risk HPV types would prevent the majority of cases of genital warts and cervical cancer, respectively.

The transmission typically occurs through the skin-to-skin anogenital contact. Increased risk for acquiring HPV has been associated with multiple sexual partners, younger age of sexual debut, failure to use condoms, and sex with uncircumcised males [10]. However, one study reported that 20% of women became infected with only one lifetime sex partner, suggesting that both partners must be sexually naïve to prevent infection [10]. Several studies have shown that the risk of infection increases substantially when initiating a new sexual relationship [5, 10–13]. The transmission of HPV infection can be blocked by latex condoms if the infected area is physically covered [14]. Nonetheless, HPV often manifests on external anogenital sites not covered by a condom, and so the latter does not prevent all infections. However, the use of a condom may reduce HPV persistence and therefore aid in the regression of HPV-associated lesions [15].

## ECONOMIC BURDEN OF HPV INFECTION

**Figure 2:**
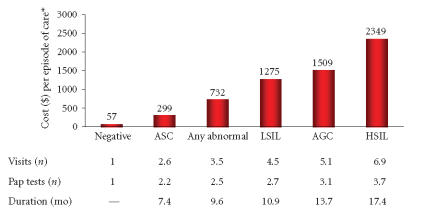
Average health care costs of cervical HPV infection [16]. *Average age adjusted to the 1998 US female population; all cost estimates were converted to 2002 dollars; ASC = atypical squamous cells; AGC = atypical glandular cells; LSIL = low-grade squamous intraepithelial lesion; HSIL = high-grade squamous intraepithelial lesion.

Annual cervical cancer screening is expensive. In a study of women enrolled in a USA health care plan, it was estimated that an average of $26,415 per 1000 women was spent on annual cervical screening and treatment for HPV-related diseases [16]. When these data are extrapolated to the general USA population, it can be estimated that $3.4 billion is spent annually on diagnosis and treatment of HPV infection and its associated cervical diseases [16]. Approximately 90% of the estimated cost can be attributed to strategies for prevention of cervical cancer, such as treatment of precancerous lesions and routine Pap tests, whereas the other 10% is spent on treatment of cervical cancer (Figure 2) [16].

HPV infection is most prevalent in adolescents and young adults, and this group also incurs the majority of HPV-associated health care costs. When it comes to HPV-related health care, women in the 20–29 year age group incurred an annual cost of $51,863 per 1000 women [16]. The estimated lifetime total medical cost of HPV infection for men and women aged 15–24 is $2.9 billion, which makes HPV the second most expensive STI after HIV [17]. In addition, when the cost of treating genital warts is analyzed by itself, it becomes apparent that this too causes a substantial economic burden. Based on an incidence of 500,000 cases of HPV infection per year, the annual total direct medical cost for treatment of anogenital warts in all age groups for the year 2000 was $167.4 million [17].

## PROPHYLACTIC VACCINES: A PREVENTATIVE STRATEGY FOR HPV INFECTION

Assembly of infectious virus, a necessary step in the HPV life cycle, involves the formation of the capsid, or outer layer, of the virion. The capsid is composed of two proteins, L1 and L2, which are expressed later in infection. The major capsid protein L1 comprises the outermost layer of the capsid and is necessary for virus structure [18]. HPV vaccine development has been considerably advanced due in part to the production of virus-like particles (VLPs). “HPV-like” VLPs, which mimic the structure of the HPV virion but do not contain genetic material and can be manufactured by exogenous expression of L1 in a variety of cell types, including bacterial, yeast, insect, and mammalian cells [18, 19]. VLPs are noninfectious and nononcogenic, making them ideal candidates for use in HPV vaccine production. VLPs are purified, concentrated, distributed into aliquots, and combined with an adjuvant [20].

Early studies with a monovalent vaccine against HPV 16 have shown that VLP vaccines induce a strong immune response against L1 in animal models [21, 22] and humoral immunity in humans [23]. Other trials demonstrated that booster doses of VLP vaccines induced protective levels of antibodies [23–25]. Furthermore, in a proof-of-principle study, the HPV 16 VLP vaccine was safe, well tolerated, and induced antibody titers to levels significantly higher those produced in response to natural infection [26]. Although this study was not sufficiently powered to assess vaccine efficacy in preventing clinical disease, vaccine recipients developed fewer cervical lesions than placebo recipients. In addition, the vaccine was 100% effective in preventing persistent infection, suggesting that VLP vaccines may help reduce the incidence of cervical cancer precursors and invasive cervical cancer [26]. Similar results have been reported with other monovalent VLP vaccines [27–29].

Immune responses to HPV infection are type-specific; therefore, vaccine efficacy can be greatly improved by combining VLPs from several types of HPV into one multivalent vaccine. Multivalent vaccines that offer protection against the most common disease-causing HPV types are in late stages of clinical development. Currently, a bivalent vaccine that protects against 2 high-risk HPV types, and a quadrivalent vaccine that protects against 2 high-risk and 2 low-risk HPV types are being tested.

To determine the efficacy, immunogenicity, and safety of a bivalent vaccine containing HPV types 16 and 18, a double-blind, placebo-controlled phase 2 trial was conducted on 1113 women (15–25 years old) with no prior history of HPV infection and normal cervical cytology [30]. In this study, women received intramuscular injections of vaccine (20 μg of each VLP plus adjuvant) or placebo (adjuvant alone) on day 1, at month 1, and at month 6, and then followed for at least 17 months. The bivalent HPV 16/18 vaccine was well tolerated, produced no vaccine-related serious adverse events, and induced a major humoral immune response to both HPV types. Furthermore, the vaccine was 90% efficacious at reducing incident infection and 100% efficacious at preventing persistent infection [30].

**Figure 3:**
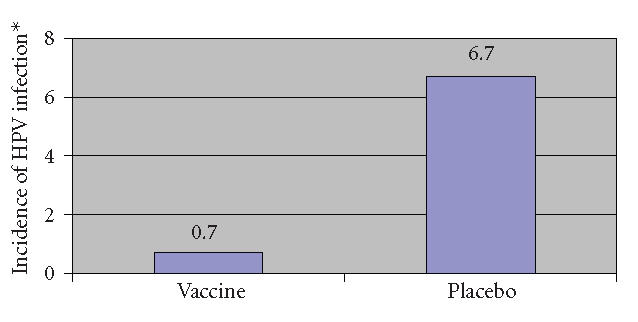
Incidence of infection or disease associated with HPV 6, 11, 16, or 18 after vaccination with a quadrivalent vaccine versus placebo (*reported as incidence per 100 women-year at risk) [31].

Including additional HPV types in vaccines would be expected to cumulatively reduce HPV-associated disease burden by preventing additional HPV infections. A quadrivalent vaccine has been developed to protect against HPV types 6, 11, 16, and 18. A double-blind, placebo-controlled phase 2 safety and efficacy trial was conducted on more than 500 women aged 16–23 years. Women who were enrolled in the trial received either the quadrivalent vaccine (20 μg each of HPV 6 and 18 VLP, and 40 μg each of HPV 11 and 16 VLP plus adjuvant) or placebo (adjuvant alone) on day 1, month 2, and month 6, and then were followed for 36 months [31]. Results of this trial showed that the quadrivalent vaccine was well tolerated, produced few serious adverse events (none of which were judged to be related to the vaccine), and stimulated the production of antibodies directed against all four HPV types. Furthermore, the vaccine reduced persistent infection in vaccine recipients by 89% and prevented 100% of clinical disease associated with HPV types 6, 11, 16, and 18 (Figure 3). Similarly high efficacy results were reported for a cohort of women who did not adhere completely to the study protocol [31].

Recently, data from the phase 3 Females United to Universally Reduce Endo-ectocervical disease (FUTURE II) clinical trial were presented. The quadrivalent HPV vaccine was 100% effective at preventing CIN 2/3, AIS, and cervical cancer associated with HPV 16 or 18 infection during two years of follow-up. The vaccine was well-tolerated and there were no vaccine-related serious adverse events [32].

## PUBLIC HEALTH BENEFITS OF HPV VACCINATION

Vaccines that provide protection against the most common disease-causing HPV types would be expected to significantly reduce the incidence of HPV-associated diseases. Reducing HPV-associated disease burden may also reduce the health care costs associated with these diseases.

Although HPV vaccines are not currently available to the public, their potential public health benefits have been reported in several mathematical modeling studies [33–35]. Sanders et al reported that if a hypothetical vaccine that was 75% efficacious at preventing high-risk HPV infection were administered to approximately two million 12-year-old girls, it would prevent 224,255 HPV infections, 3317 cases of cancer, and 1340 cervical cancer-associated mortalities in the girls' lifetimes [33]. Another study predicted that an HPV 16/18 vaccine would reduce the number of cervical cancer cases associated with the HPV 16 and HPV 18 by 95% [34]. Mathematical modeling and sexual transmission data have also suggested that both sexes should be vaccinated to provide the greatest reductions in HPV infections. For example, one population-level study predicted that a female-only HPV vaccination program would be only 68% as effective in reducing HPV prevalence as a program aimed at vaccinating both men and women [35].

Genital warts and abnormal Pap tests can also produce anxiety and emotional distress. In fact, diagnosis with genital warts is often the most anxiety-provoking outcome of HPV infection [36]. Because vaccination has shown promising results in the reduction of the disease burden associated with HPV infection, it would be expected to reduce some of the psychosocial and emotional burden as well.

A number of obstacles will need to be overcome to maximize the public health benefits of HPV vaccination. Vaccination programs must identify appropriate candidates for vaccination, establish booster requirements, and overcome potential individual, parental, and social barriers to HPV vaccine acceptance. For example, individuals may view acceptance of HPV vaccines as admitting to risky sexual behavior. Furthermore, research has shown that knowledge about HPV, an infection that many people know little about, is directly correlated to vaccine acceptance [37–39]. Parents may feel that their child is not at risk, or that vaccination would support teenage sex or encourage risky sexual behavior (ie, reduced condom use). Societal and cultural issues may include beliefs that sexually transmitted diseases are a deterrent or punishment for non-marital sexual behavior. Alternatively, many people distrust medical technology and are generally opposed to vaccines. Each of these obstacles can be overcome by widespread efforts to educate individuals and society about the prevalence of HPV and the risks associated with forgoing vaccination.

Administering HPV vaccines to populations prior to initiating sexual activity will yield the greatest health benefit. Because preadolescent and adolescent children are generally sexually naïve and develop robust immune responses to vaccines, vaccinating young adolescents is predicted to significantly reduce the incidence of HPV infection and HPV-associated diseases. In order to foster broad acceptance of HPV vaccine, public health initiatives will need to educate parents/caregivers as well as health care professionals about the risks associated with HPV infection and the benefits of vaccination.

## CONCLUSIONS

Vaccination has been widely accepted as an effective means by which infectious diseases can be prevented or eliminated. VLP vaccines that protect against infection with the most common disease-causing HPV types are currently in clinical development and early reports have suggested that HPV vaccines are highly efficacious in preventing HPV infection and HPV-associated disease. HPV vaccines will be most effective when administered prior to initiation of sexual activity and vaccination initiatives will most likely target preadolescent and adolescent populations.

## Figures and Tables

**Table 1:** Common HPV types associated with HPV-related diseases [5].

	HPV types	Manifestations

High-risk	16, 19, 31, 33, 45	Low-grade cervical changes
		High-grade cervical changes
		Cervical cancer
		Anogenital and other cancers

Low-risk	6, 11	Benign low-grade cervical changes
		Condylomata acuminata (genital warts)
